# Initial effective stress controls the nature of earthquakes

**DOI:** 10.1038/s41467-020-18937-0

**Published:** 2020-10-12

**Authors:** François X. Passelègue, Michelle Almakari, Pierre Dublanchet, Fabian Barras, Jérôme Fortin, Marie Violay

**Affiliations:** 1 Laboratoire de Mécanique des Roches, École Polytechnique Fédérale de Lausanne, Lausanne, Switzerland; 2 Centre de Géosciences, MINES ParisTECH, PSL Research University, Fontainebleau, France; 3 The Njord Centre for Studies of the Physics of the Earth, University of Oslo, 0371 Oslo, Norway; 4 École Normale Supérieure, UMR8538, 24 rue Lhomond, 75005 Paris, France

**Keywords:** Natural hazards, Geophysics, Seismology

## Abstract

Modern geophysics highlights that the slip behaviour response of faults is variable in space and time and can result in slow or fast ruptures. However, the origin of this variation of the rupture velocity in nature as well as the physics behind it is still debated. Here, we first highlight how the different types of fault slip observed in nature appear to stem from the same physical mechanism. Second, we reproduce at the scale of the laboratory the complete spectrum of rupture velocities observed in nature. Our results show that the rupture velocity can range from a few millimetres to kilometres per second, depending on the available energy at the onset of slip, in agreement with theoretical predictions. This combined set of observations bring a new explanation of the dominance of slow rupture fronts in the shallow part of the crust or in areas suspected to present large fluid pressure.

## Abstract (web summary)

The authors show that seismogenic faults can be activated by stress perturbations by all possible modes of slip independently of the frictional properties. They demonstrate, that the nature of seismicity is mostly governed by the initial stress level along the faults.

## Introduction

**Fig. 1:**
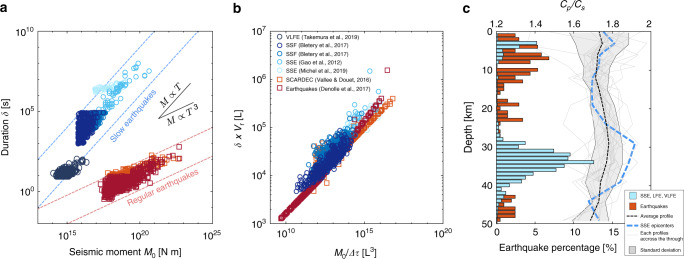
Scaling relation between the different modes of slip. **a** Scaling law for natural earthquakes observed from seismological or geodetical measurements. Blue circles correspond to slow slip events (so-called SSEs), deep low-frequency earthquakes (LFEs), very-low-frequency earthquakes (VLFEs) and slow slip fronts (SSF)^7,9,10,13,−^^15,19,62^. Regular earthquakes are presented by the red circles. The source time functions used were downloaded from the SCARDEC database^16,63^. Most of the data for regular earthquakes were taken from recent studies^62,63^. The dashed lines correspond to the regular trend deduced from natural observations for both slow (blue dashed lines) and fast earthquakes (red dashed lines)^1,7^. **b** Normalised scaling low assuming the average rupture velocity and the average static stress drop as determined in previous work for both regular^62,63^ and slow slip events^1,7,9,10,15,19^. **c** Hypocentral distribution of both slow and fast earthquakes in Japan^7,8,13–19^. Lines present the wave velocity profiles obtained from the analyses of seismic and teleseismic waves in the subducting Philippine Sea plate in the Tokai district, Japan^20,23^. The blue dashed line corresponds to the profile cross-cutting LFE hypocenters^23^. The black dashed line corresponds to the average of all grey profiles obtained along the subduction trench^23^. The grey area corresponds to the standard deviation.

Recent geophysical observations around the world have highlighted that faults release elastic strain energy stored in the wall rocks through different types of slip events. Faults generate slow rupture phenomena (~0.1–1 m/s)^1^, but also regular (~3000 m/s, also called fast ruptures) and supershear (~4200 m/s) earthquakes^1–3^. Importantly, the nature of slip along a fault seems to be variable in space and time^4^. This complexity around fault ruptures and slip behaviours results in difficulty of evaluating the seismic risk of seismogenic areas. Understanding the physical parameters and the environmental conditions controlling the rupture velocity (*V*_r_) is crucial because earthquake damage increases with this parameter^5,6^. While seismology allows estimating the size of the events and their durations^1^, the parameters controlling the nature of the slip events, as well as the reasons whether slow events obey or not similar scaling laws from regular earthquakes (Fig. 1a), remain poorly understood.

In particular, the seismic moment of regular earthquakes scales with the duration of the events as *M*_0_ ∝ *δ*^3^, whereas the seismic moment of slow ruptures, such as tremors, low-frequency earthquakes (LFE) or slow slip events (SSE), has been shown for long to scale following *M*_0_ ∝ *δ*, suggesting different propagation dynamics (Fig. 1a)^1,7–9^. However, recent seismological observations have demonstrated that, considering a single population of events presenting a large range in magnitude, the moment-duration of slow slip events scales as *M*_0_ ∝ *δ*^3^ (Fig. 1a)^10^. It is well established that for a given rupture length, the resulting slip is smaller during slow rupture phenomena than during regular earthquakes, suggesting smaller stress drop^1,7,11^. Making the hypothesis that slow slip events consist of rupture propagating circularly at a constant rupture velocity, i.e., as most regular earthquakes, we can normalise the seismic moment of each event by their average stress drops and multiply their durations (*δ*) by their average rupture velocities (*V*_r_). This normalised scaling reveals that the different moment-duration relations could just be related to variations in both stress drop and rupture speed (Fig. 1b), in agreement with recent studies^10^. This could imply three important consequences: (i) the stress drop during events is a function of the rupture velocity; (ii) since most SSEs present pulse-like behaviour, the rupture velocity increases during rupture propagation^12^; and (iii) both slow and fast earthquakes are governed by similar physics.

What are the parameters controlling the rupture velocity in nature? Along subduction interfaces, such as the Japanese trench, modern seismology has determined that the distribution of rupture phenomena is organised^7,8,13–19^. Slow rupture phenomena are generally observed at depths between 28 and 40 km, where they appear to be the dominant mode of slip, or in the shallow part of the accretionary prism, where they coexist with regular earthquakes (Fig. 1b). In some cases, these slip phenomena has been related to occur in environments presenting high seismic velocity ratios, i.e., high Poisson ratio, suggesting high fluid pressure (Fig. 1c)^20–23^. Fluid overpressure is known to play a key role in the quasi-static reactivation of faults^24^, as well as in the nucleation^25^ and the propagation of slip instabilities^26–28^. The promotion of slow slip events rather than regular earthquakes in areas presenting high fluid pressure was generally explained by an increase in the nucleation length with increasing fluid pressure, as expected by both rate-and-state and slip-weakening theories^25,29–32^. Such behaviour has been observed experimentally by the reproduction of a quasi-static rupture mode, such as stable slip^33–36^. However, these theories do not explain the quasi-dynamic propagation of slow fronts in nature or the radiation of low-frequency waves at their rupture tips. These slow fronts occur in a context where the nucleation length is smaller than the fault, suggesting that others processes inhibit the acceleration of the rupture front. From now, different possible mechanisms have been reported, such as dilatancy strengthening^37^, rate dependence of the frictional behaviour or healing front propagating behind the rupture front.

Here, we reproduce at the scale of the laboratory the complete spectrum of rupture velocities observed in nature. Our results show that when the nucleation length is within the fault length, the rupture velocity can range from a few millimetres to kilometres per second, depending on the available energy at the onset of slip. Our results are analysed in the framework of linear elastic fracture mechanics and highlight that the nature of seismicity is governed mostly by the initial stress level along the faults. Our results reveal that faults presenting similar frictional properties can rupture at both slow and fast rupture velocities.

## Results

### Experimental apparatus

The experimental setup used in this study was designed to trigger a rupture front along a critically loaded fault interface by locally increasing the pore fluid pressure. Our method allows us to study the influence of the initial stress distribution and the presence of fluid overpressure on rupture propagation. Experiments were conducted on saw-cut samples of crustal rock in tri-axial loading conditions (Supplementary Fig. 1a) that reproduce natural pressure conditions. The apparatus used in this study is a tri-axial oil medium loading cell (*σ*_1_ > *σ*_2_ = *σ*_3_) built by Sanchez Technologies. The confining pressure is directly applied by a volumetric servo-pump up to a maximum of 100 MPa. The axial stress is controlled independently by an axial piston controlled by a similar servo pump. The axial stress can reach 680 MPa on 40-mm diameter samples. Both confining and axial pressure are controlled and measured with a resolution of 0.01 MPa. Axial contraction is measured by averaging the values recorded on three capacitive gap sensors located outside of the vessel. These sensors record both the sample deformation and that of the apparatus. The resolution of these measurements is 0.1 μ. Both pressure and displacement data were recorded at the maximum sampling rate during experiments (2.4 kHz). Note that because of our sample geometry, increasing the differential stress leads to an increase in both shear and normal stresses. In addition to the record of regular mechanical data (*σ*_1_, *σ*_3_, axial strain *ϵ*_1_, radial strain *ϵ*_3_ and axial contraction), eight strain gages equally spaced at 0.8 cm and recording preferentially axial strain were glued 3 mm from the fault plane along the fault strike (Supplementary Fig. 1a). This strain gage array is used to monitor the propagation of the rupture front during episodic slip events and to image the evolution of the stress distribution profile along the fault during experiments. The local shear stress was computed from the resolved stresses accounting for the transient changes in the axial stress recorded by the strain gage array. Transient changes in the confining pressure were neglected, which is justified by the relatively high compliance of the confining medium^38^. Note that the strain gages data allow to record slip front velocity up to a maximum of 180 m/s. For faster events, we used acoustic records to track the rupture front velocity, as used in previous studies^39–41^.

### Hydraulic and frictional strength of the fault

Prior to the injection experiments, the hydraulic transmissivity of the fault was measured over the complete range of effective pressure tested using constant flow methods. The hydraulic transmissivity was estimated assuming non-linear flow lines along the fault interface^42^. The in-plane hydraulic transmissivity is estimated directly from the volumetric flux following

1 $${\zeta }_{hy}=kw=\frac{J\eta log(\frac{2a}{{r}_{0}}-1)}{B\pi \frac{{\mathrm{d}}P}{{\mathrm{d}}x}}$$

where *k* is the permeability of the fault, *w* is the fault thickness, *a* is half of the distance between the boreholes, *r*_0_ is the borehole diameter, $\frac{{\mathrm{d}}P}{{\mathrm{d}}x}$ is the imposed pressure gradient, *J* is the volumetric fluid flux, *η* is the fluid viscosity and *B* is a constant of order unity. Based on this estimate, the hydraulic transmissivity of the fault is observed to decrease from 10^−17^ to 10^−18^ m^3^ between 20 and 100 MPa effective normal stress (Supplementary Fig. 1b). These results are compatible with experimental faults presenting the same geometry^42^ and suggest a fault permeability between 10^−13^ and 10^−14^ m^2^ assuming a fault aperture ranging from 10 to 100 μ. This range of permeability is comparable to that of previous studies on similar fault geometry^42^ and initial and final roughness levels of the fault. Secondly, the peak shear strength of the fault at the onset of slip was determined at different confining pressures (30, 60 and 95 MPa). At each confining pressure, the initial pore pressure along the fault is set to 10 MPa and is regulated to remain constant during the axial loading tests up to the reactivation of the fault. The static friction of the fault is  ≈0.62 (Supplementary Fig. 1c), in agreement with Byerlee’s law^43^. In the following we present the results of 12 injection experiments. Four experiments were conducted at each confining pressure (30, 60 and 95 MPa), and each of them were conducted at the same initial pore pressure (${P}_{f}^{i}=10$ MPa).

### Influence of effective stress on the seismic behaviour

**Fig. 2:**
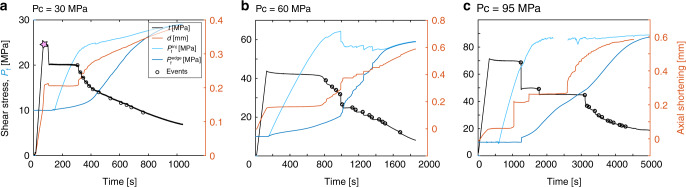
Triggering of instabilities along a critically loaded crack. Evolution of the macroscopic shear stress and of the total axial shortening recorded by external gap sensors during experiments conducted at 30 (**a**), 60 (**b**) and 95 (**c**) MPa confining pressure. The data presented in figure **a** include also the loading up to the peak strength of the fault, denoted by the pink star. Before each injection, the shear stress was first placed at 90% of the peak strength of the fault. Then, fluid was injected at a constant volume rate into the injection borehole (Supplementary Fig. 1a), up to the release of the energy stored in the system. Black, red, cyan and blue solid lines correspond to the evolution of the shear stress, the fault slip, the fluid pressure in the injection borehole and the fluid pressure in the measurement borehole, respectively. Black circles correspond to each slip instability treated in this study. The missing data in panel **c** are related to experimental maintenance of the fluid pressure system. The fluid pressure during this period is constant along the experimental fault.

Based on the reactivation criteria of the fault, we performed injection experiments along the fault preloaded at 90% of the peak shear strength of the fault (defined by the static friction coefficient). The larger the confining pressure, the larger the initial shear stress (~20, 42 and 70 MPa during experiments conducted at 30, 60 and 95 MPa confining pressure, respectively), i.e., the amount of energy available in the system (Fig. 2). Then, the fluid pressure was increased locally through a borehole (Supplementary Fig. 1a) at a constant volume rate to trigger a succession of slip events, up to the complete release of the energy stored in the system prior to the injection (Fig. 2). The fluid pressure was measured at both edge of the fault through boreholes connecting directly the pore fluid system to the fault plane. Pore pressure was measured externally using pore pressure transducers located as close as possible from the fault on the pore fluid circuit, as well as with the pressure transducers of the two servo-pump used. Note that no charge lose is observed between both transducers, suggesting that the pore pressure measured at the vicinity of the fault is probably close to the pore pressure on the fault.

Independent of the confining pressure, i.e., of the effective normal stress acting on the fault, a strong hysteresis is observed between the fluid pressure measured in the injection site and the fluid pressure measured at the opposite edge of the fault. For instance, at low confining pressure, the first reactivation is observed when the fluid pressure in the injection site reaches 23 MPa, while the fluid pressure at the opposite edge remains relatively low (*P*_f_ ≈ 12 MPa; Fig. 2a). The fluid pressure gradient generally decreases over time and with slip events due to fault reactivation during injection, which enhances the diffusion of the fluid pressure (Fig. 2 and Supplementary Fig. 2). We estimate the diffusivity enhancement along the fault during the experiment by inverting the pore pressure history measured in the observation site (see Methods section). We used a 2D diffusivity model assuming time variable diffusivity. Therefore, we were able to determine the pore pressure history on the entire fault during the experiment. Expressed in terms of the average values of shear stress and pore pressure profiles, our experimental results highlight that the fault reactivates when the average stress distribution reaches the corresponding Mohr-Coulomb failure criterion (Supplementary Fig. 3).

**Fig. 3:**
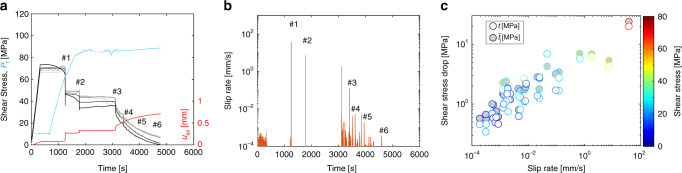
Transition from fast to slow slip events. **a** Mechanical results obtained during the experiment conducted at 95 MPa confining pressure. The evolution of the local shear stress along the fault at each strain gage location as a function of time is displayed by the grey-to-black solid lines. The evolution of the average slip along the fault is displayed by the red solid line and the evolution of the fluid pressure in the injection site by the cyan solid line. **b** Slip velocity burst associated with each slip instability. The peak slip velocity reached during instability decreases over time, i.e., with an increasing number of events and with the progressive release of the initial shear stress. Numbers displayed in **a** and **b** refer to events for which the slip front propagation is presented in Fig. 4. **c** Scaling relation between the shear stress drop versus average slip rate obtained using the average stress profile recorded by strain gages (grey circles) or pressure sensors (white circles).

**Fig. 4:**
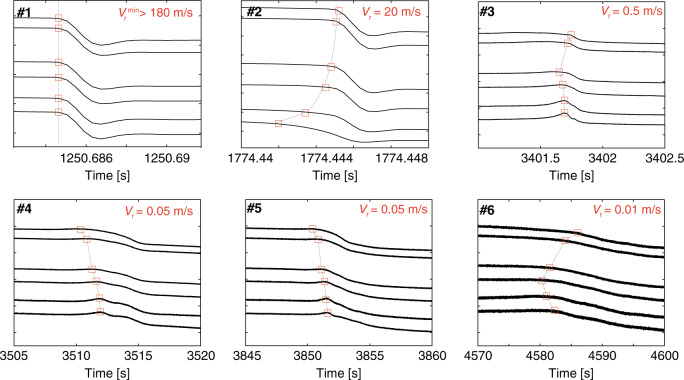
Propagation of rupture along the interface during slip events. The rupture velocity is estimated using an equidistant strain gage array along the fault. The average rupture velocity is computed using the average travel time for the rupture front. The numbers displayed at the top left corner of each plots refer to the events presented in Fig. 3a, b.

Remarkably, a transition between fast to slow slip events is observed as the injection progresses, and the average shear stress decreases on the fault (Fig. 3a and Supplementary Fig. 4). For each fast or slow release of stress, we computed an average slip (*u*) and slip velocity (*V*_s_) within the resolution of our system. Both *u* and *V*_s_ increase with increasing stress drop associated with each event (Fig. 3b, c), as observed in previous studies^38^. Second, using a strain gage array, we tracked the slip front associated with each slip event (Fig. 4). Our experimental results show that the events that propagated at the highest stress level present the highest rupture velocities, up to values close to the shear wave velocity of the bulk material. Subsequent rupture propagations, induced at lower stress levels, present slower rupture velocities, from one metre to a few millimetres per second (Fig. 4). These slow rupture velocities are in agreement with natural observations^1,7–9,11^.

At the scale of our experiments, a strong correlation is observed between the state of stress prior to the onset of a slip event and the rupture velocity^44^. Large initial shear stress seems to promote fast slip events, while low values in shear stress seems to promote the propagation of slow rupture front. Note that the rupture velocity seems independent of the nucleation location, since we observed slow slip front nucleating at both central part or edges of the fault. The same behaviour was observed for fast rupture front in previous studies^38^.

## Discussion

**Fig. 5:**
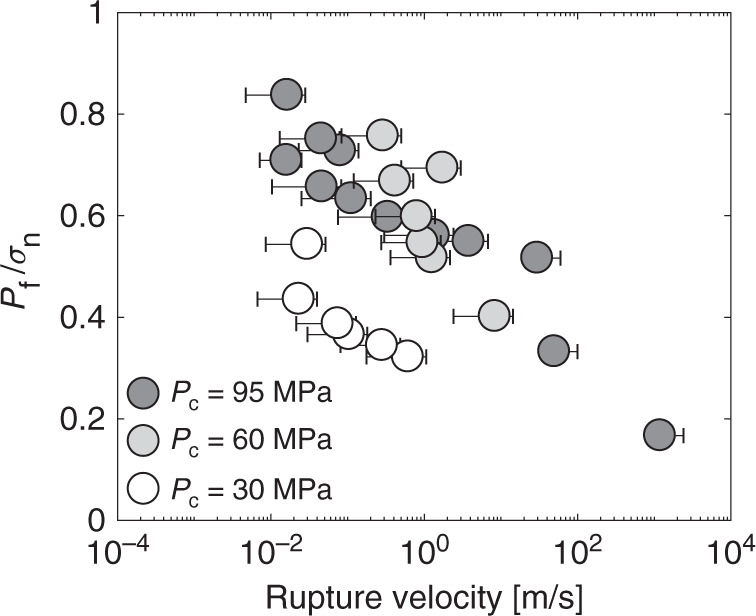
Influence of the ratio between the fluid pressure and the normal stress acting on the fault at the onset of fault slip on rupture velocity. For each confining pressure, the larger the ratio lambda, the smaller the stress drop and the smaller the rupture velocity during the instability.

**Fig. 6:**
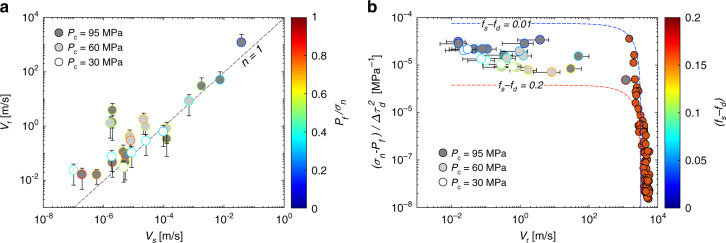
Control of the nature of the seismicity. **a** Scaling relation between the rupture velocities and the slip velocities reached during each slip event. White, grey and dark grey correspond to slip events observed at 30, 60 and 95 MPa confining pressures, respectively. The colour bar displays the ratio between the average fluid pressure and the average normal stress along the fault at the onset of the slip events. **b** Influence of the stress parameters derived from Eq. (9) on the rupture velocity achieved during slip events. White, grey and dark grey symbols correspond to slip events observed at 30, 60 and 95 MPa confining pressures, respectively. Red symbols correspond to dry experiments conducted on Westerly granite^38^. Blue and red dashed lines correspond to LEFM predictions using strength drops of 0.01 and 0.2, respectively.

For each confining pressure tested, the rupture velocity seems to increase with the decrease in the ratio between the average fluid pressure and the average normal stress (*λ* = *P*_f_/*σ*_n_) acting on the fault at the onset of propagation (Figs. 5 and 6a). However, note that a small change in fluid pressure can induce a large change in rupture speed, suggesting that fluid pressure may not be the dominant parameters. While this trend depends also slightly on the confining pressure, all data collapse when comparing the average rupture velocity to the average slip velocities reached during each instability (Fig. 6a). The slip velocity increases linearly with increasing rupture velocity achieved during the event, a behaviour that is predicted by linear elastic fracture mechanics (LEFM)^45,46^. This relation was also observed from natural observations during slow slip events^47^.

Regarding the slip mode, as state previously, we observed a correlation between *V*_r_ and *λ*, but mostly with the average initial shear stress profile (${\bar{\tau }}_{0}$) at the onset of the slip event. Assuming that the nucleation length is systematically smaller than the length of the fault, a condition required for the propagation of both slow and fast rupture in fracture mechanics and friction theories, our experimental results can be analysed in the framework of linear elastic fracture mechanics. From these conditions and the observation that the slip front process zone remains much smaller than the specimen dimensions, the rupture velocity is expected to be a function of the energy available at the rupture tip. Indeed, following LEFM predictions for a dynamic shear crack, *V*_r_ can be expressed following *V*_r_ = *C*_R_(1 − *G*_c_/Γ) for sub-Rayleigh ruptures, where *C*_R_ is the Rayleigh wave velocity, *G*_c_ is the fracture energy required to advance the slip front and *Γ* is the energy release rate. Assuming that *G*_c_ depends only on the effective normal stress before the rupture, this relation can be written as a function of the initial stress acting on the fault:

2 $${V}_{{\mathrm{r}}}={C}_{{\mathrm{R}}}\left(1-\frac{({\sigma }_{n}-{P}_{{\mathrm{f}}})}{\Delta {\tau }_{d}^{2}}\frac{\Omega }{\pi L}{E}^{* }\right)$$

where Δ*τ*_d_ is the dynamic stress drop during rupture, *L* is the length of the crack, and E^*^ is the dynamic Young’s modulus (E^*^ = *E*/(1 − *ν*^2^) in-plane strain). Ω is a term describing the effect of frictional weakening with slip. For a linear slip-weakening model of friction, Ω = (*f*_s_ − *f*_*d*_)*d*_c_, with *d*_c_ being the characteristic slip for frictional weakening and *f*_s_ and *f*_d_ are respectively the static and dynamic friction coefficients. This relation sheds light directly on the dependence between the initial state of stress acting on the fault and the rupture velocity observed. Considering that Ω is not a function of the initial stress, the rupture velocity is expected to decrease with increasing fluid pressure (Methods section, Eq. (10)) or with decreasing initial stress acting on the fault (${\bar{\sigma }}_{n}$ and ${\bar{\tau }}_{0}$ in our experiments) because both lead to a decrease in Δ*τ*_d_ during events^48^ (i.e., to an increase in the ratio $({\sigma }_{{\mathrm{n}}}-{P}_{{\mathrm{f}}})/{(\Delta {\tau }_{{\mathrm{d}}})}^{2}$). We now compare our experimental results to LEFM predictions^31^. First, we compute the stress terms in Eq. (2) for each event using direct measurements. For each confining pressure tested, *V*_r_ increases with decreasing $({\sigma }_{{\mathrm{n}}}-{P}_{{\mathrm{f}}})/\Delta {\tau }_{{\mathrm{d}}}^{2}$, although some exceptions exist (Fig. 6b). Note that our experimental results are strongly consistent with the theoretical predictions computed for two different values of strength drop (*f*_s_ − *f*_d_) and assuming *d*_c_ ≈ 10 μm^31^ and *L* = *L*_f_ = 0.08 cm (*L*_f_ being the length of the experimental fault). Both experimental results and theoretical predictions highlight that a low stress level promotes the propagation of slow rupture phenomena. Moreover, note how the supershear ruptures observed in previous experiments under dry conditions^38^ extend the scaling predicted by the Eq. (2) for sub-Rayleigh velocities^49^. The good collapse of the supershear data is explained by the fact that relations similar to the ones used in Eq. (2) (see also Methods section) also exist in the supershear regime, except that the functional *g*(*V*_r_) for supershear velocities also depends on the initial loading conditions^49^.

The dynamic fracture theory behind Eq. (2) predicts that a nucleated rupture front quickly accelerates toward fast propagation speed approaching *c*_R_. So why do slow ruptures not accelerate to seismic wave velocities in nature? Different strengthening mechanisms buffering rupture acceleration have been proposed in the literature^50^, including dilatancy^28,51^ and the heterogeneity of the fault zone that combines velocity-weakening patches surrounded by stable velocity-strengthening regions. Interestingly, these two scenarios are not expected to prevail in the context of our experiments. Small pressure variations are indeed observed during the rupture propagation and the frictional response is expected to be uniform along the homogeneous pre-cut interface. The co-existence of fast and slow ruptures during the same experiment can then be explained by a non-monotonic frictional response of the interface^52,53^, i.e. a transition from velocity-weakening friction at low slip velocity to velocity-strengthening friction at faster velocity. In this context, the emergence of a significant stress drop is prevented once the fault operates at low level of shear stress, which only enables quasi-static rupture propagation driven by the slip gradient developping between the locked and the (slowly) slipping portion of the fault. This explanation is supported by recent theoretical and numerical studies^54^ as well as by the evolution of Δ*τ*_d_ observed in the experiments for lower values of shear stress and higher fluid pressures. Finally, in nature, the low initial stress conditions may not be enough to extend the rupture length *L*, limiting and buffering the rupture front velocity (Eq. (9)).

Our new results demonstrate that seismogenic faults can be activated by stress perturbations by all possible modes of slip independently of the frictional properties. The slip mode depends only on the initial stress acting along the fault ahead of the rupture tip, i.e., the energy stored along the fault. Note that in nature, large values of *P*_f_ may imply small values of ${\bar{\tau }}_{0}$ because of the slow far field loading rate compared to the rate of fluid pressure accumulation. For instance, assuming certain hypotheses (Methods section, Eq. (11)), slow rupture velocities (*V*_r_ < 0.1 m/s) are expected to occur when faults are subjected to an initial effective normal stress of 10 MPa, which implies almost lithostatic fluid pressure at depth, in agreement with natural observations^23,55,56^. Our results explain why slow ruptures are promoted in over-pressurised areas or at shallow depths (Fig. 1c), where the stress is expected to remain low during the seismic cycle. Our results also support the spatio-temporal variability of the mode of slip in nature since the stress acting on faults evolves both in time and in space. However, recent observations on post seismic response of repeating earthquakes^57,58^ have suggested that the rupture process could be influenced by friction and stressing rate, rather than by coseismic stress. According to LEFM theory and the experiments shown here, the rupture velocity also depends on friction (on the difference between static and dynamic friction). However, we cannot show here a possible influence of the stressing rate on rupture speed since this parameter was constant during the experiments. This issue would require more investigation. Note also that this interpretation of repeating earthquake data relies on a model involving a seismogenic asperity embedded in a creeping fault, leading to complex redistribution of coseismic stresses. In this framework, the level of stress on the asperity prior to rupture propagation is not really constrained by observations, and may affect the rupture process as well.

## Methods

### Sample preparation

Cylindrical samples (diameter: 40 mm, length: 90 mm) were cored from andesite blocks from the Déhaies quarry, located in Guadeloupe (France)^59^. This andesite was found to have a density of 2690 kg/m^3^ and a porosity ranging from 1.1 and 2.3%, in agreement with a previous study^59^. This rock was chosen due to (i) anticipated future exploitation of the reservoir by a geothermal project and (ii) the negligible permeability of the bulk of the intact specimen (<10^−21^ m^−2^)^59^, which ensures purely in-fault fluid diffusion during the injection experiments.

Prior to experiments, the rock cylinders were saw-cut to create an experimental fault at an angle of 30^o^ with respect to *σ*_1_ (the principal stresses are denoted *σ*_1_ > *σ*_2_ = *σ*_3_). The fault surfaces were roughened first with grinder and then with coarse sandpaper (grit number P240,  ≈50 μm roughness) using ethanol to avoid frictional heating during sample preparation. All experiments were conducted on a fault surface presenting the same initial geometry and roughness. To induce injection along the artificial fault interface, boreholes were drilled at both edges of the fault (Supplementary Fig. 1a). The bottom borehole was used as the injection site, while the top borehole was used only as a measurement site (Supplementary Fig. 1a).

### Numerical modelling of the pore pressure distribution

The model assumes a homogeneous diffusivity along the fault, which is modelled as an ellipsis. Neumann boundary conditions are assumed at the edge of the ellipsis in agreement with our experimental conditions (i.e., no fluid flow outside of the ellipsis). Because of the low permeability of the bulk of the sample (≈10^−21^ m^−2^ (Supplementary Fig. 1b), we assume purely in-fault fluid diffusion between the injection site and the measurement borehole. We assume that the hydraulic diffusivity along the fault is spatially constant but changes over time. The pressure is then estimated using the diffusion equation

3 $$\frac{\partial p(x,y,t)}{\partial t}=D\left(\frac{{\partial }^{2}p(x,y,t)}{\partial {x}^{2}}+\frac{{\partial }^{2}p(x,y,t)}{\partial {y}^{2}}\right)$$

The 2D diffusion equation is evaluated using the finite volume method. The fault is discretised into 64 cells and 32 cells in the length and width of the fault, respectively. The stability of the system is ensured since

4 $$\Delta t=\frac{\Delta {x}^{2}\Delta {y}^{2}}{2D(\Delta {x}^{2}+\Delta {y}^{2})}=\frac{\Delta {x}^{2}}{4D}$$

where Δ*t* and Δ*x* and Δ*y* are the time and spatial steps, respectively. Note that in our calculation, we assume that Δ*x* = Δ*y*. The pressure at the injection and measurement boreholes are then used to invert the spatial evolution of the fluid pressure along the entire fault plane. To improve our estimates, we allow an increase in diffusivity over time. The results regarding the evolution of the hydraulic diffusivity during the experiments are beyond the scope of this paper but are partially presented in Supplementary Fig. 2a, which presents the comparison between the experimental measurements and the prediction, as well as the evolution of the hydraulic diffusivity required to invert the experimental data. This numerical modelling provides an estimate of the fluid pressure along the fault during the experiments, which is used to estimate the average fluid pressure at instability in the manuscript (Fig. 4).

### Slip front equation of motion from LEFM

The dynamic energy release rate for a mode II crack in the sub-Rayleigh regime can be written as^45^:

5 $${\Gamma }_{{\mathrm{II}}}(L,{V}_{{\mathrm{r}}})=g({V}_{{\mathrm{r}}}){G}_{{\mathrm{II}}}(L,{V}_{{\mathrm{r}}}=0).$$

In the equation above, *g*(*V*_r_) is a universal function of the rupture velocity, and *G*_II_(*L*, *V*_r_ = 0) is the energy release rate for a static crack of the same length *L*, which can be expressed as^60^:

6 $${\Gamma }_{{\mathrm{II}}}(L,{V}_{{\mathrm{r}}}=0)=\chi {(\Delta {\tau }_{{\mathrm{d}}})}^{2}\pi L\frac{1}{2{E}^{* }}$$

where *χ* is a dimensionless variable in the range of unity accounting for the geometry of the crack, Δ*τ*_d_ is the dynamic stress drop and E^*^ = *E*/(1 − *ν*^2^) is the plane strain condition. The crack tip energy balance implies that the dynamic energy release rate should always equal the fracture energy *G*_c_ during the rupture. Using Freund’s approximation^45^ *g*(*V*_r_) = (1 − *V*_r_/*C*_R_) and Eq. (5), the energy balance leads to the following crack tip equation of motion:

7 $${V}_{{\mathrm{r}}}(L)={C}_{{\mathrm{R}}}\left(1-\frac{{G}_{{\mathrm{c}}}}{{\Gamma }_{{\mathrm{II}}}(L,{V}_{{\mathrm{r}}}=0)}\right).$$

In the context of frictional rupture, the fracture energy can be expressed as

8 $${G}_{{\mathrm{c}}}=\frac{1}{2}({\sigma }_{{\mathrm{n}}}-{P}_{{\mathrm{f}}})\Omega$$

with Ω being a generic function describing frictional weakening with slip. For the linear slip-weakening model^60^, Ω = (*f*_s_ − *f*_d_)*d*_c_ with *f*_s_ and *f*_d_ as the static and dynamic friction coefficients, respectively, and *d*_c_ the slip-weakening distance. For rate-and-state models of friction, $\Omega =\alpha {\mathrm{ln}\,}^{2}({V}_{{\mathrm{s}}}/{V}_{0})$^61^, with *V*_s_ and *V*_0_ being the slip velocity behind and ahead of the front, respectively, and *α* a constant depending only on the rate-and-state parameters.

Combining Eqs. (6), (7), (8) and taking *χ* = 1, the rupture velocity can be expressed as a function of the initial effective normal stress, the dynamic stress drop and the length of the crack, which are directly measured through our experiments:

9 $${V}_{{\mathrm{r}}}={C}_{{\mathrm{R}}}\left(1-\frac{({\sigma }_{n}-{P}_{{\mathrm{f}}})}{\Delta {\tau }_{{\mathrm{d}}}^{2}}\frac{\Omega }{\pi L}{E}^{* }\right).$$

This equation can then be directly related to the effective normal stress by considering that Δ*τ*_d_ = (*σ*_n_ − *P*_f_)(*f*_0_ − *f*_d_), where *f*_0_ is the background friction coefficient along the fault. Based on this hypothesis, the rupture velocity can be approximated by

10 $${V}_{{\mathrm{r}}}={C}_{{\mathrm{R}}}\left(1-\frac{1}{({\sigma }_{{\mathrm{n}}}-{P}_{{\mathrm{f}}}){({f}_{{\mathrm{0}}}-{f}_{{\mathrm{d}}})}^{2}}\frac{\Omega }{\pi L}{E}^{* }\right)$$

and the effective stress leading to a sub-Rayleigh rupture velocity can be expressed as follows:

11 $$({\sigma }_{{\mathrm{n}}}-{P}_{{\mathrm{f}}})=\left(\frac{1}{(1-\frac{{V}_{{\mathrm{r}}}}{{C}_{{\mathrm{R}}}}){({f}_{{\mathrm{0}}}-{f}_{{\mathrm{d}}})}^{2}}\frac{({f}_{{\mathrm{s}}}-{f}_{{\mathrm{d}}})\kappa }{\pi }{E}^{* }\right)$$

where *κ* = *d*_c_/*L* (≈10^−5^ in our experiments). Note that if we assume that *d*_c_ is a linear function of *L*, which is assumed in seismology, this last relation can be used to estimate the initial effective stress that leads to a given rupture velocity independent of the crack length. For instance, assuming fixed values for (*f*_0_ − *f*_d_) = 0.1 and (*f*_s_ − *f*_d_) = 0.5, slow rupture velocities (*V*_r_ < 0.1 m/s) should be promoted when faults are subjected to an initial effective normal stress of ~10 MPa, which implies lithostatic fluid pressure at depth.

## Supplementary information

Supplementary Information

## Data Availability

The data necessary to reproduce the figures presented in this paper are available in the supplementary files. The raw data that support the findings of this study are available from the corresponding author upon reasonable request.

## Source data

Source Data
