# Supplementary material for: Initial effective stress controls the nature of earthquakes
Source: Nat Commun. 2020 Oct 12;11:5132. doi: 10.1038/s41467-020-18937-0 (PMC7552404; doi:10.1038/s41467-020-18937-0)
Supplement: Supplementary file 1 — Supplementary Information [file 41467_2020_18937_MOESM1_ESM.pdf]

Supplementary Information for

Initial effective stress controls the nature of earthquakes

by Passelegue et al., 2020

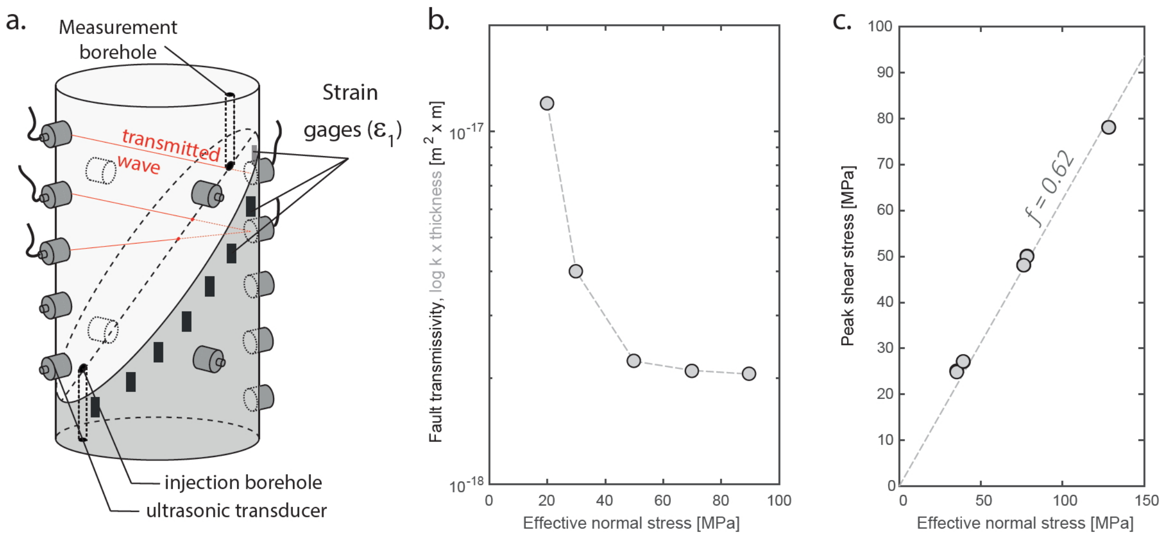

Supplementary Figure 1.

Experimental setup and properties of the fault. (a.) Schematic of the sample assembly. The length of the fault is 8 cm along strike. Injection is conducted in the bottom sample through a borehole reaching the fault surface. The evolution of the fluid pressure is measured at the opposite edge of the fault through a measurement borehole. Strain gages are located along one side of the fault. Acoustic sensors were used in a passive way to monitor acoustic wave radiations (b.) Evolution of the hydraulic transmissivity along the fault as a function of the effective normal stress. (c.) Static peak strength of the fault.

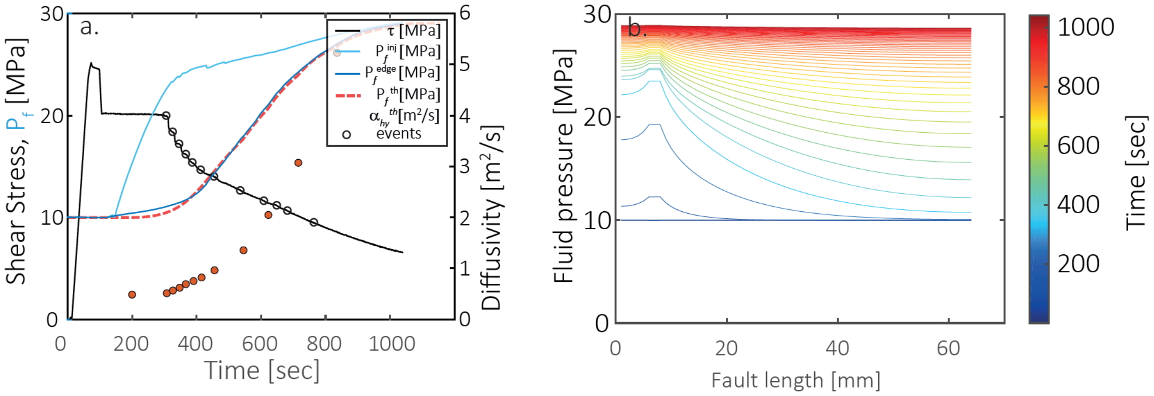

Supplementary Figure 2.

Numerical results. (a.) Numerical and experimental pressure at the injection and measurement borehole. Black solid line refers to the evolution of the shear stress during the experiment, and black circles denote slip events. Light blue and dark blue solid lines refer to the fluid pressure measured at the injection and measurement boreholes, respectively. Dashed red line presents the result of the inverted pore pressure using the change in average hydraulic diffusivity represented by the red circles. (b.) Evolution of the pore pressure profile along the strike of the fault during the injection. Time interval is 42 seconds.

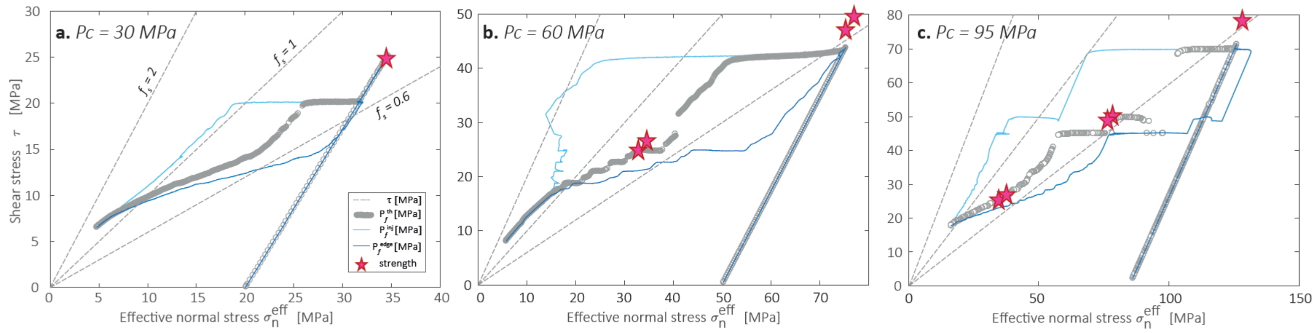

Supplementary Figure 3.

Mohr diagrams presenting the onset of fault reactivation conditions in experiments conducted at 30, 60 and 90 MPa confining pressure (a., b. and c., respectively). Light and dark blues solid lines correspond to the effective stress paths computed using the fluid pressure in the injection and measurement boreholes, respectively. Grey circles correspond to the effective stress paths estimated using the average pore pressure profile inverted using the numerical model. Dashed grey lines correspond to different values for the static friction. Pink stars corresponds to the peak strength of the fault measured during dedicated experiments.

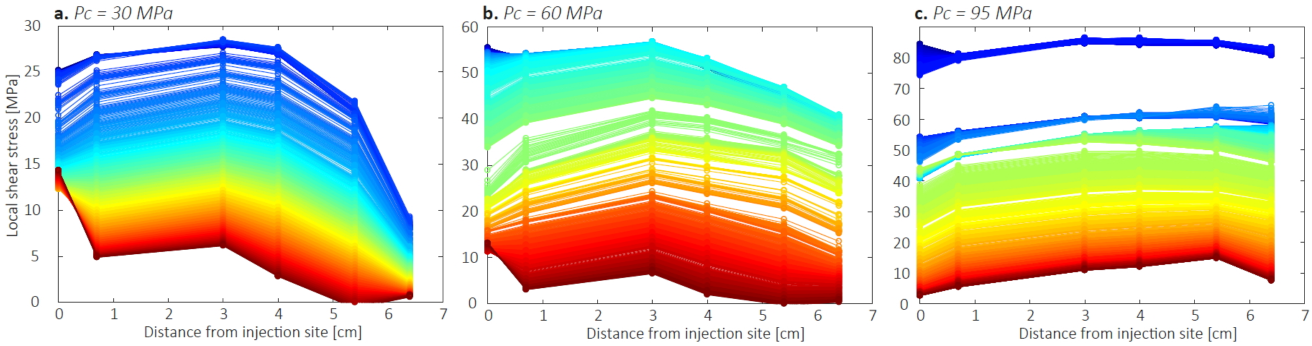

Supplementary Figure 4.

Evolution of the shear stress along the fault during experiments conducted at 30, 60 and 90 MPa confining pressure (a., b. and c., respectively). Shear stress is estimated using strain gages considering no change in confining pressure, due to regulation during the injection. The colour bar displays the normalized time series (blue is the initial stress and red the stress at the end of the experiments).
